# Synergistic Antibacterial Interaction of Geraniol and Biogenic Silver Nanoparticles on Methicillin-Resistant *Staphylococcus aureus*

**DOI:** 10.3390/plants14071059

**Published:** 2025-03-29

**Authors:** Isabela Madeira de Castro, Camila Antunes, Camila Cristina Valentim, Laís Fernanda de Almeida Spoladori, Helena Tiemi Suzukawa, Guilherme Ferreira Correia, Gislaine Silva-Rodrigues, Paulo Henrique Guilherme Borges, Guilherme Bartolomeu-Gonçalves, Mariana Luiza Silva, Marcelle de Lima Ferreira Bispo, Rayanne Regina Beltrame Machado, Celso Vataru Nakamura, Gerson Nakazato, Phileno Pinge-Filho, Eliandro Reis Tavares, Lucy Megumi Yamauchi, Sueli Fumie Yamada-Ogatta

**Affiliations:** 1 Programa de Pós-graduação em Microbiologia, Universidade Estadual de Londrina, Londrina 86055-900, Paraná, Brazil; isabela.mcastro@uel.br (I.M.d.C.); lais.spoladori@gmail.com (L.F.d.A.S.); helena.tiemi.suzukawa@uel.br (H.T.S.); guilhermeferreiracorreia@gmail.com (G.F.C.); gislaine.srodrigues@uel.br (G.S.-R.); pauloguilhermeph@gmail.com (P.H.G.B.); cvnakamura@uem.br (C.V.N.); gnakazato@uel.br (G.N.); pingefilho@uel.br (P.P.-F.); tavares.eliandro@uel.br (E.R.T.); lionilmy@uel.br (L.M.Y.); 2 Laboratório de Biologia Molecular de Microrganismos, Universidade Estadual de Londrina, Londrina 86055-900, Paraná, Brazil; camila.antunes110702@gmail.com (C.A.); camila.cristina@uel.br (C.C.V.); guilherme.bartolomeu@uel.br (G.B.-G.); 3 Programa de Pós-graduação em Fisiopatologia Clínica e Laboratorial, Universidade Estadual de Londrina, Londrina 86038-350, Paraná, Brazil; 4 Laboratório de Síntese de Moléculas Medicinais, Departamento de Química, Centro de Ciências Exatas e da Terra, Universidade Estadual de Londrina, Londrina 86055-900, Paraná, Brazil; mariana.luiza@uel.br (M.L.S.); mlfbispo@uel.br (M.d.L.F.B.); 5 Laboratório de Inovação Tecnológica no Desenvolvimento de Fármacos e Cosméticos, Universidade Estadual de Maringá, Maringá 87020-900, Paraná, Brazil; raymachado6@hotmail.com; 6 Laboratório de Imunopatologia Experimental, Departmento of Imunologia, Parasitologia e Patologia Geral, Universidade Estadual de Londrina, Londrina 86055-900, Paraná, Brazil; 7 Departamento de Medicina, Pontifícia Universidade Católica do Paraná, Londrina 86067-000, Paraná, Brazil

**Keywords:** biofilm inhibition, bactericidal activity, molecular docking, monoterpenoid, staphyloxanthin inhibition, synergism, toxicity

## Abstract

Since ancient times, plants have been used in folk medicine to treat different diseases. Plants offer exceptional chemical diversity with a wide range of biological activities, and have therefore been the most promising sources for the discovery and development of drugs, including antimicrobial agents. This study reports the antibacterial effect of geraniol (GER), alone and in combination with biogenic silver nanoparticles (bioAgNPs), produced using the aqueous extract of *Trichilia catigua* bark, against planktonic and sessile cells of methicillin-resistant *Staphylococcus aureus* (MRSA), one of the main opportunistic and potentially fatal human pathogens. GER had a time-dependent bactericidal effect on planktonic cells, impairing the cell membrane integrity. In addition, GER inhibited the staphyloxanthin production, and molecular docking analyses supported the in silico affinity of GER to dehydrosqualene synthase (CrtM) and 4,4′-diaponeurosporen-aldehyde dehydrogenase (AldH), which are key enzymes within the pigment biosynthesis pathway in *S. aureus*. GER treatment increased the sensitivity of MRSA to hydrogen peroxide killing. GER displayed synergism with bioAgNPs against planktonic and sessile cells, inhibiting bacterial adhesion and the viability of biofilms formed on abiotic surfaces. MRSA planktonic and sessile cells treated with GER or GER/bioAgNPs displayed severe morphological and ultrastructural alterations. Notably, neither GER nor its combination caused in vitro and in vivo toxicity in mammalian cells and *Galleria mellonella* larvae, respectively. These findings suggest that the combination of GER/bioAgNPs may be a promising strategy to control MRSA infections.

## 1. Introduction

*Staphylococcus aureus* is one of the most common members of the human microbiota [1]; however, it is also one of the most frequent opportunistic human pathogens, causing a wide variety of clinical manifestations [2] in both healthcare and community settings all over the world [3,4,5,6]. The genome plasticity of this bacterium allows for efficient adaptability to different host niches [7]. Contributing to this adaptation, *S. aureus* has acquired resistance to almost all clinically available antibacterial agents [8]. Worryingly, within the pathogen–drug combinations, methicillin-resistant *S. aureus* (MRSA) has been identified as the leading cause of death attributed to antimicrobial resistance [9], representing a major global therapeutic challenge. Along with the resistance of planktonic cells, biofilm formation represents an additional threat to the antimicrobial therapy of staphylococcal infections. Notably, sessile cells within biofilms display enhanced resistance to most antimicrobials and have the ability to evade host defenses, resulting in persistent and difficult-to-treat infections [10,11,12]. Particularly, in wound infections, biofilms contribute to delayed wound-healing and skin repair, and may act as precursors of systemic infections [13]. Given this alarming scenario, the World Health Organization has classified MRSA as a high-priority bacterial pathogen for the research and development of new control strategies [14].

The combined inhibitory effect of two or more compounds may be a promising approach to overcome antimicrobial resistance [15,16]. In fact, several antimicrobial combinations have been clinically used for the treatment of infectious diseases [16]. For instance, the combination of trimethoprim with sulfonamides was reported in 1968 [17] and has been used thereafter in the treatment of microbial infections. Overall, the combination of two or more compounds aims at achieving synergistic antimicrobial effects, thereby improving their effectiveness; suppressing the emergence of antimicrobial resistance; minimizing compound toxicity to the host; and expanding the spectrum of antimicrobial coverage during therapy [18].

The search for antimicrobial molecules isolated from plants is one of the main approaches to developing novel therapeutic strategies for microbial infections. In fact, several phytochemicals have been identified with multi-target effects, exhibiting antimicrobial, anti-inflammatory, antioxidant, and anticancer activities [19,20]. One such example is geraniol (GER), a monoterpenoid component of essential oils from different plant species [20]. Regarding antimicrobial effects, several studies have reported the growth-inhibitory activity of GER against planktonic cells of different fungal and bacterial species, including MRSA [20,21]; however, the antibiofilm activity of this compound remains underexplored.

Plant extracts or their phytochemicals have also been widely used for the biosynthesis of metallic nanoparticles [22,23,24]. Given their unique properties, biogenic silver nanoparticles (bioAgNPs) have proven to be versatile compounds with promising applications across various fields, including biomedicine [24]. Historically, silver salts have been used as topical medicines due to their antimicrobial properties, especially in wound care [25].

Currently, in addition to wound care, bioAgNPs are utilized in a diverse range of applications, such as cosmetics, food shelf-life extension, implanted medical devices (catheters), and targeted drug delivery systems [24]. Indeed, several studies have shown that bioAgNPs can inhibit the growth of diverse microbial species, including those exhibiting antimicrobial resistance, such as MRSA strains [23,26,27]. Due to their size, AgNPs display enhanced molecular interactions with target microorganisms, with minimal toxic effects to mammalian cells [28]. Additionally, antioxidant, antidiabetic, anticancer, anti-inflammatory, and wound-healing properties have been attributed to bioAgNPs [24].

Although the inhibitory effect of GER alone [29,30,31] and bioAgNPs alone [23,32,33,34,35] on the growth of MRSA strains has been reported previously, their combined antimicrobial activity has not been reported to date. Therefore, herein we describe the antibacterial effect of the combination of GER and bioAgNPs against planktonic and sessile (biofilm) cells of MRSA. The biogenic AgNPs (bioAgNPs) were obtained using the aqueous extract of *Trichilia catigua* Adr. Juss bark.

## 2. Results and Discussion

### 2.1. Geraniol Displays Synergistic Interaction with Biogenic Silver Nanoparticles Against Planktonic Cells of Methicillin-Resistant Staphylococcus aureus

In this study, the inhibitory effect of GER and bioAgNPs was initially screened by the broth microdilution assay, and the results revealed the susceptibility of all strains to both compounds. A minimum inhibitory concentration (MIC) value of 625 μg/mL for geraniol was identified for all MRSA strains, regardless of their SCC*mec* types (Supplementary Table S1), indicating moderate activity, according to Sartoratto et al. [36]. The minimum bactericidal concentration (MBC) values were 1250 μg/mL for four strains (including the reference MRSA BEC 9393 strain) and 2500 μg/mL for six strains. The MBC/MIC ratios were two or four, indicating a bactericidal effect [37] (Table 1). These values are consistent with the MIC and MBC ranges of GER against MRSA reported in the literature, which were 512 to 4250 μg/mL [29,30,31] and 1000 to 1024 μg/mL [29,30], respectively. The bactericidal effect of GER may result from its hydrophobic nature, facilitating the interaction with the membrane lipids, resulting in structural disruption and increased permeability [20].

The bioAgNPs used in this study were obtained from the reduction of silver nitrate by the aqueous extract of *T. catigua* A. Juss. bark, a plant from the *Meliaceae* family, which is native to Latin America and part of the Brazilian flora [40]. According to a technical report, these water-soluble bioAgNPS exhibited spherical morphology with dimensions of approximately 90–100 nm, which was further confirmed using dynamic light scattering [41]. The MIC and MBC values of bioAgNPs for all MRSA strains were 8.43 µg/mL and 16.87 µg/mL, respectively, and the MBC/MIC was 2, indicating a bactericidal effect [37] (Table 1). The antibacterial effect of silver nanoparticles obtained from plant-mediated green synthesis, using plant extracts or phytochemicals against MRSA, has also been previously described. In this regard, prior studies have reported MIC and MBC values ranging from 1.56 µg/mL to 1250 µg/mL and 50 µg/mL to 2500 µg/mL, respectively [23,32,33,34,35]. Variations in MIC and MBC values may occur due to the size, shape, charge, and the capping agent of the bioAgNPs [22,23,24], from which released silver ions (Ag^+^) mediate the bactericidal effect. Particularly, Ag^+^ can slowly penetrate into bacterial cells of *Staphylococcus* spp., increasing the production of reactive oxygen species (ROS). These reactive molecules interact with nucleic acids, proteins, and lipids, resulting in the inhibition of DNA replication and protein synthesis, as well as cell membrane damage [23,28].

**Figure 1:**
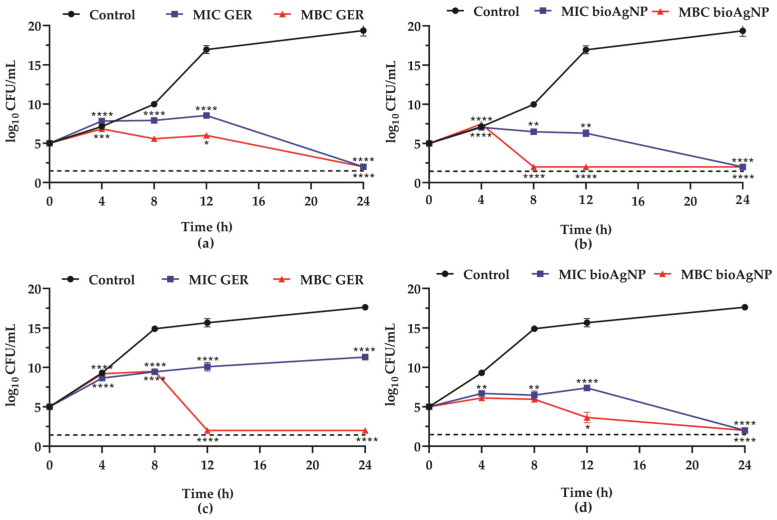
Antibacterial activity of geraniol (GER, **a**,**c**) and biogenic silver nanoparticles (bioAgNPs, (**b**,**d**)) against methicillin-resistant *Staphylococcus aureus*. Time–kill kinetics of MRSA BEC 9393 (**a**,**b**) and MRSA PSA1 (**c**,**d**) incubated with minimum inhibitory (MIC) and minimum bactericidal (MBC) concentrations. The log_10_ CFU/mL values represent the mean ± the standard deviation from three independent experiments. The dashed lines indicate a 99.9% (3 log_10_) reduction in CFU/mL counts. * (*p* < 0.05), ** (*p* < 0.01), *** (*p* < 0.001),**** (*p* < 0.0001) compared to the initial inoculum.

To elucidate the bactericidal nature of GER and bioAgNPs, and to assess the time–kill kinetics of MRSA, the growth curves of planktonic cells, in the presence of each compound individually, were monitored for 24 h at 37 °C. Given the MIC and MBC values of GER and bioAgNPs, MRSA BEC 9393 (Figure 1a,b) and MRSA PSA1 (Figure 1c,d) were selected for this analysis (thereafter, these strains were named BEC 9393 and PSA1, respectively). Overall, both compounds at their MIC values inhibited the growth of MRSA strains over time compared to untreated control cells. Interestingly, except for PSA1 in the presence of GER (Figure 1c), a 3-log_10_ reduction in colony-forming unit (CFU)/mL counts of the initial inoculum (corresponding to 99.9% reduction) was observed for both compounds at their respective MIC and MBC values after a 24 h incubation period (Figure 1a,b,d). For PSA1, a slight increase in the CFU counts was observed after 4 h of incubation, which remained constant throughout the period analyzed, after treatment with GER at its MIC. However, following the incubation period (24 h), a significant reduction (*p* < 0.0001) in CFU counts was observed compared to the untreated control. Conversely, a significant reduction (*p* < 0.0001) in CFU counts was observed after 12 h of incubation at its MBC, compared to the initial inoculum (Figure 1c).

**Figure 2:**
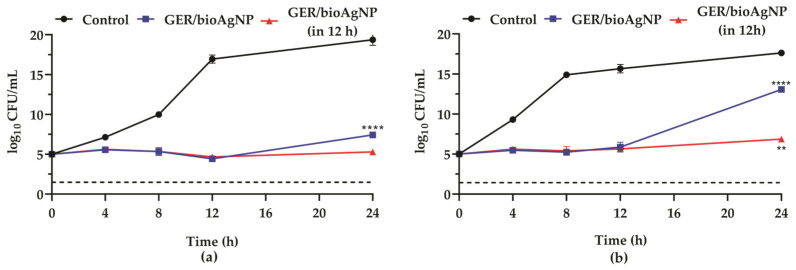
Antibacterial interaction of geraniol (GER) and biogenic silver nanoparticles (bioAgNPs) against methicillin-resistant *Staphylococcus aureus*. Time–kill kinetics of MRSA BEC 9393 (**a**) and MRSA PSA1 (**b**) incubated with GER and bioAgNPs at synergistic concentrations. The red line indicates the addition of fresh medium containing GER/bioAgNPs after 12 h of incubation. The log_10_ CFU/mL values represent the mean ± the standard deviation from three independent experiments. The dashed lines represent a 99.9% (3 log_10_) reduction in the CFU/mL counts. ** (*p* < 0.01), **** (*p* < 0.0001) in comparison to the initial inoculum.

Thereafter, the antibacterial effect of GER combined with bioAgNPs on planktonic cells of MRSA strains was evaluated by checkerboard assay [38], and the results are shown in Table 1. According to the fractional inhibitory concentration index (FICI = 0.5), the simultaneous addition of GER and bioAgNPs displayed synergistic antibacterial activity. The combination of both compounds at synergistic concentrations inhibited bacterial growth for up to 12 h of incubation, after which bacterial cells resumed growth, increasing the CFU counts, as illustrated in Figure 2a,b. However, the addition of fresh medium containing the compounds at the synergistic concentrations after 12 h maintained the growth inhibition of MRSA (Figure 2a,b). These results indicate that the combination of GER and bioAgNPs at synergistic concentrations results in a bacteriostatic effect, and, ideally, a subsequent dose should be administered approximately 12 h after the initial dose. Although the combination of GER and bioAgNPs results in a bacteriostatic effect, a four-fold reduction in the MIC values of both compounds was observed, compared to the effect of each compound alone. Indeed, randomized controlled trials have reported equivalent or even superior efficacy of bacteriostatic agents in the treatment of several microbial infections [42]. Further investigations, including pre-clinical and clinical studies, can corroborate the application of this combination as a novel alternative for the treatment of MRSA infections.

**Figure 3:**
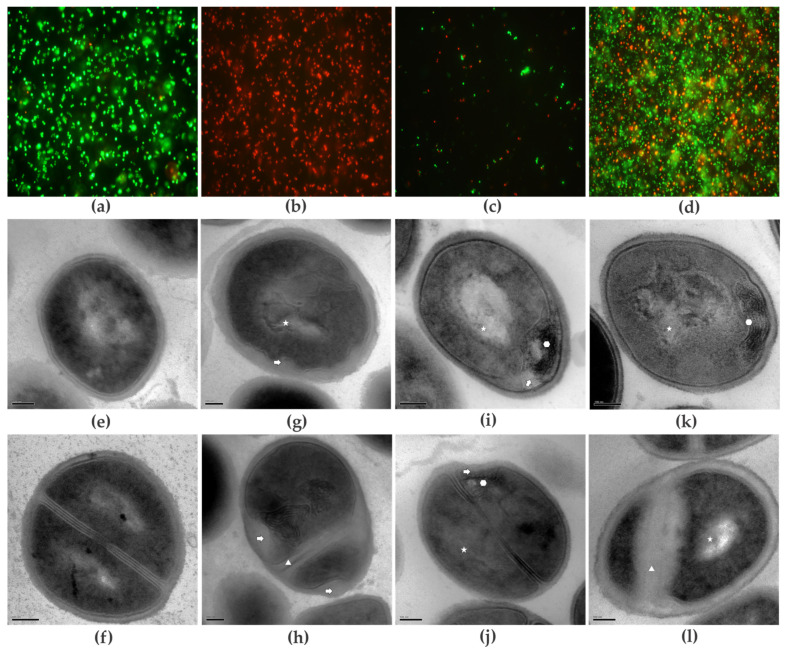
Effect of geraniol (GER) and biogenic silver nanoparticles (bioAgNPs), alone or in combination (GER/bioAgNPs), on the morphology and viability of methicillin-resistant *Staphylococcus aureus* planktonic cells. Bacterial cells were incubated with or without the MICs of the individual compounds, or at synergistic combination for 1 h (cell viability) and 12 h (transmission electron microscopy—TEM). Cell viability analysis (**a**–**d**) of MRSA BEC 9393 after differential labeling with SYTO 9™ and propidium iodide. The green fluorescence represents metabolically active bacteria, and red fluorescence indicates metabolically inactive bacteria with damaged membranes (1000× magnification). Representative TEM images (**e**–**l**) of MRSA BEC 9393. Arrow: deformation of the cell envelope; star: decreased cytoplasmic density; triangle: asymmetric formation of the cell division septum; hexagon: concentric myelin-like structures. (**a**,**e**,**f**) Untreated MRSA (control); (**b**,**g**,**h**) treatment with GER MIC (625 µg/mL); (**c**,**i**,**j**) treatment with bioAgNPs MIC (8.43 µg/mL); (**d**,**k**,**l**) treatment with the GER/bioAgNPs combination (156.25/2.10 µg/mL). Bars: 100 nm.

The effect of GER, bioAgNPs, and their combination on BEC 9393 cell membrane integrity was evaluated by microscopy after differential cell labeling with the fluorescent probes, SYTO 9™ and propidium iodide (Figure 3a–d). The untreated planktonic cells (Figure 3a) exhibited green fluorescence, indicating the presence of metabolically active cells with intact membranes. Conversely, at the MIC values of GER (Figure 3b) and bioAgNPs (Figure 3c), the presence of red-fluorescent cells was observed, indicating metabolically inactive cells with damaged membranes. Cells treated with GER and combined with bioAgNPs at the synergistic concentrations displayed diffuse green or reddish fluorescence, indicating the simultaneous presence of metabolically active and dead cells (Figure 3d). These data corroborate the bactericidal nature of each compound individually, and the bacteriostatic nature of the compound combination, as previously determined through the time–kill assays.

Morphological and ultrastructural alterations of BEC 9393 planktonic cells induced by GER and bioAgNPs, administered individually at MIC values and in combination at the synergistic concentrations, were also observed by transmission electron microscopy (TEM, Figure 3e–l). The control untreated cells exhibited typical spherical morphology, showing a compact cell wall with homogeneous electron density in the cytoplasm, and formation of the cell division septum (Figure 3e,f). GER (625 µg/mL) caused significant changes in the BEC 9393 morphology and ultrastructure after 12 h of incubation, such as cell envelope deformation, areas with decreased cytoplasmic electron density, and asymmetric formation of the cell division septum (Figure 3g,h). Changes in the appearance of the cytoplasmic content, and more electron-dense regions with a concentric myelin-like appearance, were observed in BEC 9393 after incubation with bioAgNPs (8.43 µg/mL, Figure 3i,j). GER (156.25 µg/mL) combined with bioAgNPs (2.10 µg/mL) resulted in changes similar to those observed with the compounds alone (Figure 3k,l).

Although the mode of action of the GER/bioAgNPs interaction was not the main focus of our study, some evidence can contribute to the understanding of this antibacterial effect, such as the following: (i) monoterpenoids are capable of crossing the cell wall and permeabilizing the plasma membrane [43]; (ii) monoterpenoids, such as thymol [44] and GER [45,46], inhibit the production of staphyloxanthin, a membrane-bound pigment that increases the rigidity of cell membrane [47,48] and may be related to increased resistance to antimicrobial cationic molecules, including human neutrophil defensin-1, a synthetic platelet peptide congener (RP-1), polymyxin B, and daptomycin [47]; and (iii) Gram-positive bacteria are more resistant to AgNPs than Gram-negative bacteria [49]. In light of these data, we hypothesize that GER may decrease the biosynthesis of staphyloxanthin, thus altering membrane fluidity and increasing permeability to bioAgNPs. To investigate this hypothesis, we evaluated the effect of GER, bioAgNPs, and their combination on MRSA pigments.

### 2.2. Geraniol Interferes with the Pigmentation of Methicillin-Resistant Staphylococcus aureus

**Figure 4:**
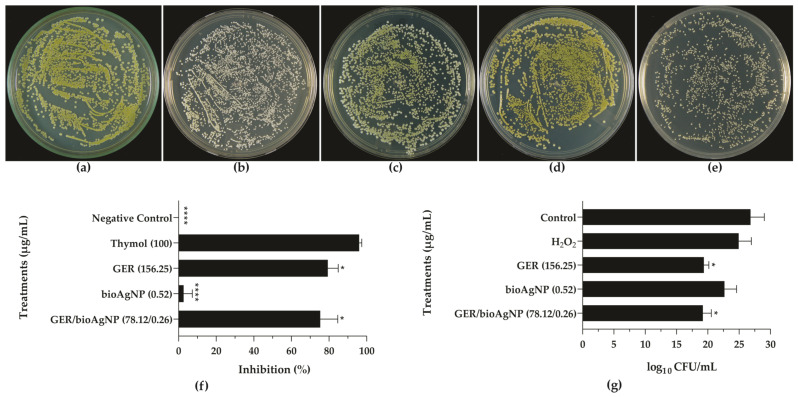
Effect of geraniol (GER) and biogenic silver nanoparticles (bioAgNPs), alone or in combination (GER/bioAgNPs), on staphyloxanthin production (**a**–**f**) and sensitivity to hydrogen peroxide (**g**) in methicillin-resistant *Staphylococcus aureus* planktonic cells. For qualitative analyses, MRSA BEC 9393 was cultivated on Mueller–Hinton agar (MHA) in the absence ((**a**), negative control) or presence of 100 µg/mL thymol ((**b**), positive control), 156.25 µg/mL GER (**c**), 0.52 µg/mL bioAgNPs (**d**), and 78.12/0.26 µg/mL GER/bioAgNPs (**e**). For quantitative analyses, the pigment production was determined after methanol extraction (**f**). Sensitivity of MRSA BEC 9393 to hydrogen peroxide was also assessed (**g**). Bars graphs represent the MRSA CFU counts after a 3-h incubation in the absence (control) or presence of 0.2% hydrogen peroxide. H_2_O_2_ (control) represents MRSA without previous treatment with GER and/or bioAgNPs. **** (*p* < 0.0001), * (*p* < 0.05), compared to thymol (positive control) and H_2_O_2_ control, respectively.

Staphyloxanthin, the main carotenoid pigment of *S. aureus,* is responsible for its characteristic yellowish or orange coloration [50], and is produced by most *S. aureus* isolates in certain growth media [51]. The ability of GER and bioAgNPs to inhibit staphyloxanthin production was analyzed in this study using two approaches. For qualitative assessment, the BEC 9393 strain was inoculated onto Mueller–Hinton agar (MHA) containing subinhibitory concentrations of the compounds alone and combined. The selected sub-MICs were employed since they did not inhibit the growth of bacterial cells (Supplementary Figure S1). Yellow colonies were observed on plates without the compounds (growth control, Figure 4a), and white colonies were identified on the media containing thymol (100 μg/mL, Figure 4b), a known inhibitor of staphyloxanthin production [44]. Notably, GER also inhibited this production, as shown in Figure 4c. In contrast, bioAgNPs exhibited no such effect, as evidenced by the unaltered yellowish color of the visualized colonies (Figure 4d). Furthermore, the treatment with GER/bioAgNPs also inhibited pigment production by BEC 9393 cells (Figure 4e). To further validate the effect of GER and bioAgNPs on staphyloxanthin, a quantitative analysis of carotenoid pigment production was performed following methanol extraction. As expected, thymol resulted in 100% inhibition of the pigment production, while GER led to a 79.3% inhibition at the tested concentration. A slight reduction (2.6%) in the pigment production was observed upon treatment with bioAgNPs. Consistent with the findings obtained using GER, the combination resulted in a significant (*p* < 0.05) reduction in the pigment production (Figure 4f).

In addition to their effect on bacterial plasma membranes, the carotenoid pigments of *S. aureus* exhibit antioxidant activity [47,52] and contribute to greater survival on abiotic surfaces [53]. Due to its antioxidant properties, staphyloxanthin plays a role in the evasion of *S. aureus* from host immune defenses [47]. Specifically, staphyloxanthin conferred resistance to *S. aureus* against ROS-mediated neutrophil killing, enhancing the bacterial virulence in a mouse subcutaneous infection model [52]. To evaluate the capacity of the compounds and the combination to sensitize BEC 9393 to ROS, we performed a hydrogen peroxide killing assay. As shown in Figure 4g, GER, alone or combined with bioAgNPs, caused a significant reduction (*p* < 0.05) in bacterial CFU counts after incubation with 0.2% hydrogen peroxide, supporting that this monoterpenoid interfered with bacterial survival under ROS exposure.

To further elucidate the observed inhibition of staphyloxanthin by the monoterpenoid, a molecular docking analysis was carried out, using two enzymes from the pigment biosynthesis pathway. The staphyloxanthin biosynthetic pathway is controlled by the *crt*OPQMN operon. Dehydrosqualene synthase (CrtM) catalyzes the condensation of two molecules of farnesyl diphosphate into dehydrosqualene, followed by subsequent steps catalyzed by 4,4′-diapophytoene desaturase (CrtN), 4,4′-diaponeurosporene oxidase (CrtP), 4,4′-diaponeurosporenoate glycosyltransferase (CrtQ), and acyltransferase CrtO, leading to the formation of 4,4-diapophytoene, 4,4-diaponeutosporene, 4,4-diaponeurosporenic acid, glucosyl-4,4-diaponeurosporenic acid, and staphyloxanthin, respectively [54]. The 4,4′-diaponeurosporen-aldehyde dehydrogenase (AldH) of *S. aureus* has been identified to catalyze the oxidation of 4,4′-diaponeurosporen-4-al to 4,4′-diaponeurosporenoic acid [55,56]. These enzymes promote the functional group addition and the formation of double bonds, resulting in the final structure of staphyloxanthin (Supplementary Figure S2). Inhibition of one or more of these enzymes can, therefore, reduce and/or inhibit the production of staphyloxanthin by *S. aureus*. Indeed, a previous study showed that thymol, a monoterpene alcohol found in essential oil from various plants, inhibits the production of staphyloxanthin. This effect seems to be mediated by staphyloxanthin interactions with the amino acid residues Thr118 and Phe120 within CtrM. Furthermore, spectroscopic analysis revealed a significant reduction of all of the metabolic intermediates of the staphyloxanthin biosynthetic pathway after MRSA incubation with thymol [44]. Given that only the three-dimensional structures of CrtM and AldH are currently available in the Protein Data Bank (PDB), these enzymes were utilized to predict the potential mechanism of action of GER [46,57].

**Figure 5:**
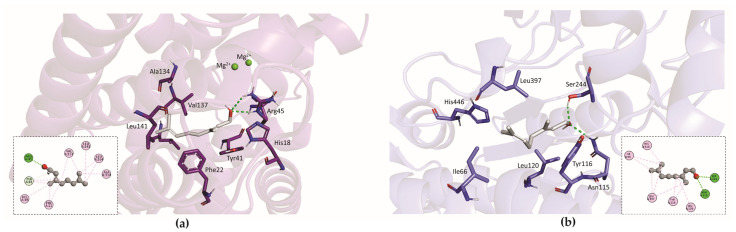
Representation of molecular interactions of geraniol (GER) with *Staphylococcus aureus* CrtM and AldH enzymes of the staphyloxanthin biosynthesis pathway. (**a**) Interaction of GER with specific amino acid residues of CrtM, wherein purple sticks represent amino acid residues; light gray sticks represent GER; dashed green lines correspond to hydrogen bonds; light green lines represent unconventional hydrogen bonds; and pink circles represent hydrophobic interactions. (**b**) Interaction of GER with specific amino acid residues of AldH, wherein blue sticks represent amino acid residues; light gray represents GER; dashed green lines correspond to hydrogen bonds; and pink circles represent hydrophobic interactions.

CrtM possesses two key active sites for the conversion of farnesyl diphosphate (FPP) to presqualene diphosphate (PSPP), which is the precursor of staphyloxanthin (Supplementary Figure S3). At site 1 (S1), where FPP binds and is ionized to form a primary carbocation, magnesium ions and residues such as arginine and tyrosine are responsible for removing the carbocation-S1 complex. This carbocation subsequently translocates to site 2 (S2) to react with the prenyl diphosphate present at this site to form PSPP. This active site is characterized by increased hydrophobicity and contains phenylalanine, alanine, and valine [58]. Although GER does not interact with the magnesium ions at S1 of the CrtM enzyme, it forms hydrogen bonds with Arg45 and His18, alongside important hydrophobic interactions with Tyr41 and other residues, including Ala134, Val137, and Leu141 (Figure 5a). Elghali et al. [46] also investigated the interaction of GER with a different CrtM structure (PDB ID: 2FNP). Consistent with our results, they reported that GER did not interact with magnesium ions, but interacted with aspartate and arginine residues via hydrogen bonding. Hydrophobic interactions were also observed along the chain.

The active site of AldH comprises several residues, including Tyr116, Ser244, Phe456, Ile66, and several proline and alanine residues. These residues bind to the NAD(P)+ cofactor and catalyze the aldehyde oxidation reaction [56]. Through the hydroxyl present in the GER structure, it forms hydrogen bonds with Ser244 and Asn115. Moreover, GER forms hydrophobic interactions with other residues, such as Ile66 and Tyr116 (within the catalytic pocket), as well as Leu397, His446, and Leu120 (Figure 5b).

Comparing the two potential targets of GER, it is clear that the monoterpenoid is more likely to inhibit AldH than CrtM. Although GER exhibits favorable interactions with CrtM residues, it does not interact with the magnesium ions essential for enzyme activity. In contrast, it interacts very similarly with the substrate 4,4′-diaponeurosporen-4-al, mainly through hydrogen bonds with Ser244 and the other hydrophobic residues.

### 2.3. Geraniol, Alone or Combined with Biogenic Silver Nanoparticles, Inhibits the Adhesion and Biofilm Formation of Methicillin-Resistant Staphylococcus aureus on Abiotic Surfaces

Microbial biofilms consist of cells tightly adhered to a surface and embedded in a self-produced extracellular polymeric matrix. Biofilms provide microorganisms with protection against predators and environmental stressors, such as those caused by nutrient availability, variations in pH and temperature, and the presence of xenobiotics [10]. This mode of growth also represents a key strategy for the colonization and pathogenesis of various microorganisms, including MRSA strains.

**Figure 6:**
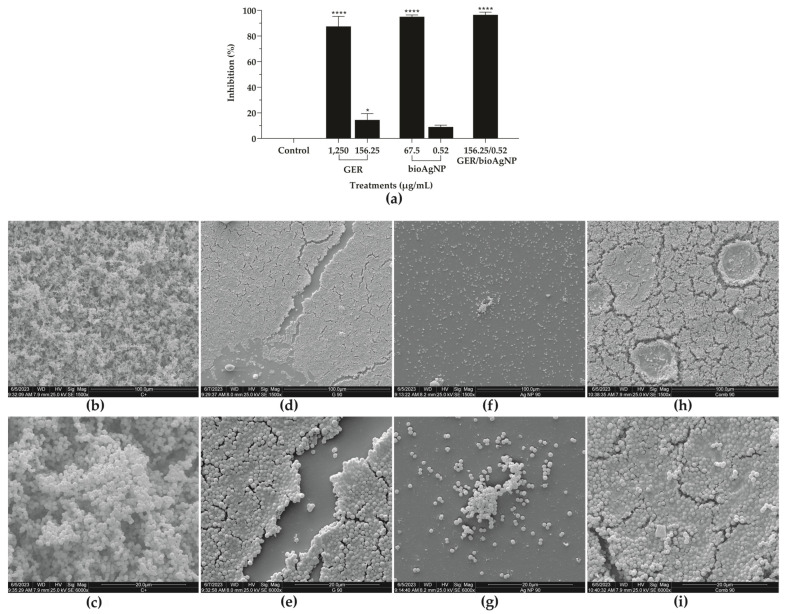
Effect of geraniol (GER), biogenic silver nanoparticles (bioAgNPs), or their combination (GER/bioAgNPs) on metabolic activity, morphology, and ultrastructure of 24 h biofilms of methicillin-resistant *Staphylococcus aureus* formed on polystyrene surfaces. (**a**) The 24 h biofilms of MRSA BEC 9393 were incubated in the absence (control) or presence of compounds, individually, at their SMIC_90_ (GER, 1250 µg/mL and bioAgNPs, 67.5 µg/mL) and at their synergistic SMIC_90_ (GER, 156.25 µg/mL and bioAgNPs, 0.52 µg/mL), and combined (GER/bioAgNPs, 156.25/0.52 µg/mL). The metabolic activity of sessile cells was determined by the MTT reduction assay. Values are expressed as the mean ± standard deviation from three independent experiments. * *p* < 0.05, **** *p* < 0.0001, compared to untreated control. (**b**–**i**) Scanning electron microscopy (SEM) micrographs of 24 h biofilms. (**b**,**c**) Untreated control; (**d**,**e**) treatment with 1250 μg/mL GER; (**f**,**g**) treatment with 67.5 μg/mL bioAgNPs; (**h**,**i**) treatment with 156.25/0.52 μg/mL GER/bioAgNPs.

The inhibitory effect of GER alone [31,45,46] and bioAgNPs alone [32,34,59,60] on MRSA adhesion and biofilms has been reported previously. In this study, we evaluated the antibiofilm activity of GER combined with bioAgNPs on 24 h biofilms of MRSA strains formed on polystyrene surfaces. First, we determined the SMIC_90_ (the lowest concentration capable of inhibiting 90% of the metabolic activity of sessile cells) of both compounds alone for all MRSA strains, and the results are shown in Table 2. The SMIC_90_ values of GER and bioAgNPs ranged from 625 to 1250 µg/mL and 16.87 to 67.5 µg/mL, respectively. The simultaneous addition of both compounds to pre-formed (24 h) biofilms resulted in a synergistic interaction (FICI ranged from 0.12 to 0.49), reducing the SMIC_90_ values of GER and bioAgNPs by 16-fold and at least 32-fold, respectively. To explore the antibiofilm effect of the combination, 24 h biofilms of BEC 9393 were incubated with GER and bioAgNPs alone at concentrations that displayed synergism (Figure 6a). A slight reduction (14.5%) in the metabolic activity of sessile cells was observed in the presence of GER (156.25 µg/mL). In addition to this, BioAgNPs at 0.52 µg/mL did not impact the viability of these cells. Conversely, a 96.5% reduction in the metabolic activity of sessile cells was observed after treatment with the synergistic combination.

Scanning electron microscopy (SEM) analyses were performed to visualize the morphological and ultrastructural alterations of BEC 9393 biofilms after treatment with GER, bioAgNPs, or their combination. A typical biofilm architecture, a three-dimensional structure consisting of a dense network of spherical cells and water channels, was observed in untreated 24 h biofilms of the MRSA strain formed on a polystyrene surface (Figure 6b,c). The treatment with GER (SMIC_90_) caused the disruption of the aforementioned structure (Figure 6d,e). Moreover, the treatment with bioAgNPs (SMIC_90_) significantly reduced the biomass of sessile cells (Figure 6f,g). Similar to the treatment with GER alone, the simultaneous addition of the plant compound with bioAgNPs resulted in the disruption of the biofilm architecture, along with a reduction in stacking layers and the presence of spherical cells embedded within amorphous substances (Figure 6h,i).

**Figure 7:**
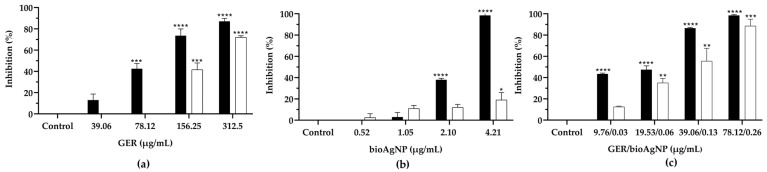
Effect of subinhibitory concentrations of geraniol (GER, (**a**)) and biogenic silver nanoparticles (bioAgNPs, (**b**)), alone or in combination (GER/bioAgNPs, (**c**)), on methicillin-resistant *Staphylococcus aureus* adhesion (black bars) and biofilm formation (white bars) on polystyrene surfaces. For adhesion assays, MRSA BEC 9393 was incubated with the compounds for 4 h. For biofilm formation experiments, after the adhesion of MRSA BEC 9393, fresh medium containing the compounds was added, and the system was incubated for a further 24 h. The metabolic activity of sessile cells was determined by the MTT reduction assay. * (*p* < 0.05), ** (*p* < 0.01), *** (*p* < 0.001), **** (*p* < 0.0001) when compared to the control.

We also evaluated whether GER and bioAgNPs, alone or in combination, could inhibit the adhesion of bacterial cells on polystyrene, as this event can trigger biofilm formation on different surfaces, as well as favor colonization of several niches and the establishment of infection [11,12,61]. GER or bioAgNPs, at subinhibitory concentrations (0.5 to 0.0625 × MIC), caused a dose-dependent inhibition of BEC 9393 planktonic cells’ adherence. Compared to the untreated control, reductions in cell adherence to the polystyrene surface, ranging from approximately 13 to 87% for GER and 3 to 98% for bioAgNPs, were observed (Figure 7a,b). Notably, all subinhibitory concentrations of the GER/bioAgNPs combination significantly reduced (*p* < 0.0001) BEC 9393 adherence to the polystyrene surface (Figure 7c). Afterward, we evaluated the effect of the compounds, alone or combined and at subinhibitory concentrations (0.5 to 0.0625 × MIC), on the biofilm formation of BEC 9393. Both compounds inhibited biofilm formation by BEC 9393. However, significant differences (*p* < 0.05) in the metabolic activity of the treated biofilms, compared to the untreated control, were observed only at higher concentrations [156.25 and 312.5 µg/mL of GER (Figure 7a); and 4.21 µg/mL of bioAgNPs (Figure 7b)]. Similarly, a dose-dependent inhibition of the biofilm formation was observed for the GER/bioAgNPs combination at subinhibitory concentrations (Figure 7c).

### 2.4. Geraniol Combined with bioAgNP Does Not Cause Toxicity to HaCat Cells and Galleria mellonella Larvae

Determining the pharmacokinetic and toxicity profiles of a drug candidate is a crucial stage in the development of novel pharmaceutical formulations. These characteristics contribute to minimizing the risk of adverse effects and therapeutic failure during clinical trials [62]. In silico analyses of absorption, distribution, metabolism, excretion, and toxicity (ADME-Tox) indicated that GER possesses drug-like properties and potential for oral administration. Moreover, no mutagenic or hepatotoxic effects were predicted [21]; in fact, GER is classified as “generally recognized as safe” (GRAS) by the Flavoring Extract Manufacturers’ Association [63], and was approved as a food flavoring agent by the US Food and Drug Administration in 2023 [64]. Conversely, a remarkable drawback of AgNPs relies on their risk of adverse effects. The concentration, composition, and dimension of AgNPs are the main properties associated with their toxic effects. In fact, as the AgNP size decreases, the surface-to-volume ratio increases, resulting in greater reactivity and toxicity [22,23,24]. However, AgNPs synthesized using plant extracts display reduced toxicity compared to those produced by chemicals or physical processes. This is mainly due to the presence of coating molecules that enhance the stability of nanoparticles, and that, in general, have their own biocompatibility with biological systems [22].

**Figure 8:**
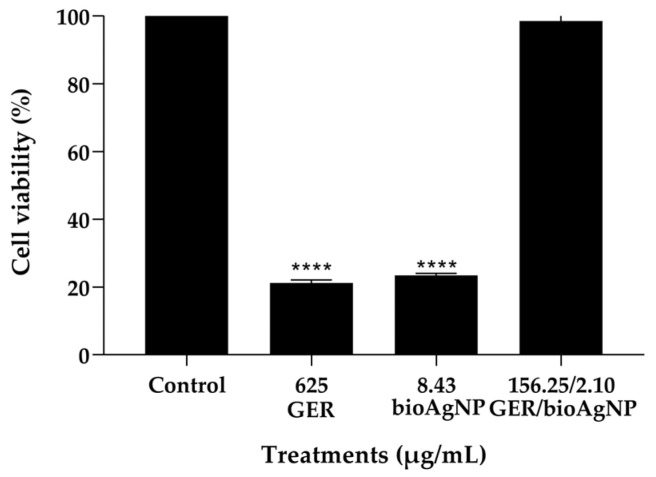
Effect of geraniol (GER) and biogenic silver nanoparticles (bioAgNPs) on metabolic activity of HaCaT cells. Cell viability of HaCat cells after 24 h treatment with GER MIC (625 µg/mL), bioAgNPs MIC (8.43 µg/mL), and the GER/bioAgNPs synergistic concentrations (156.25/2.10 µg/mL). **** *p* < 0.0001 when compared to the untreated (control) cells.

We assessed the toxicity of the compounds, alone (at MIC) or combined (at the synergistic concentration), on the viability of HaCat cells (human keratinocyte cell line) using the MTT reduction assay. Keratinocytes are the main constituents of the epidermis and play an important role in the skin’s innate immune response to microbial infections, including *S. aureus* infections [65]. After a 24 h incubation period, 21.24% and 23.49% of cells were metabolically active in the presence of GER (625 µg/mL) and bioAgNPs (8.43 µg/mL), respectively. However, at the synergistic combination (GER = 156.25 µg/mL, bioAgNPs = 2.10 µg/mL), approximately 98.5% of the cells exhibited metabolic activity (Figure 8), indicating minimal toxicity to these mammalian cells.

Another essential stage in the search for novel antimicrobial compounds consists of evaluating their toxic effects on the host. Mammalian models are important tools for assessing the pharmacological efficacy of novel compounds. Nevertheless, these models are expensive, laborious, and demand strict ethical guidelines for their use in research [66]. Based on the principles of the 3Rs (Replacement, Reduction, and Refinement) that regulate the use of mammals in scientific procedures [67], we utilized the larvae of the greater wax moth, *Galleria mellonella* (Lepidoptera: Pyralidae), to evaluate the toxicity of GER and bioAgNPs, alone or combined. The larvae of this insect can be easily maintained under laboratory conditions, and their use requires fewer ethical considerations than mammalian models [66]. The immune system of *G. mellonella* shares similarities with the human innate defense, allowing studies of the virulence and pathogenesis of different microbial species [68]. Furthermore, this invertebrate has been successfully used as a simple and cost-effective model for studying the preliminary in vivo antimicrobial activity and toxicity of several compounds [21,66,68].

In this study, a survival rate of 100% was observed in the control group (PBS-treated) and all groups treated with GER and/or bioAgNPs over five days, indicating that these compounds are not toxic to larvae at the tested concentrations (Supplementary Figure S4). A study using adult male Sprague-Dawley rats showed that administration of GER (50 mg/kg, emulsified in anhydrous glycerol) resulted in approximately 270 µg/mL of the monoterpenoid in the blood, and 2.5 µg/mL in the cerebrospinal fluid, measured 30 and 60 min after oral administration, respectively. However, a rapid elimination rate from these sites was also observed, highlighting the need for developing formulations with a controlled release of GER for clinical application [69]. Considering these results, it is important to note that the GER/bioAgNPs combination reduced the MIC of both compounds compared to the use of each compound individually. Since the concentration of 156.25 µg/mL (lower than that found in rat blood) is capable of inhibiting both planktonic and sessile MRSA cells, this reinforces its potential for the development of novel strategies for controlling infections caused by this bacterium. Further studies are required to evaluate the safety and therapeutic potential of GER combined with bioAgNPs for the treatment of MRSA infections in mammals.

## 3. Materials and Methods

### 3.1. Chemicals and Culture Media

Geraniol (GER, trans-3,7-dimethyl-2,6-octadien-1-ol, purity ≥98%), 3-(4,5-dimethylthiazol-2-yl)-2,5-diphenyltetrazolium bromide (MTT), dimethyl sulfoxide (DMSO), Tween^®^ 80, methanol, thymol (2-isopropyl-5-methylphenol), menadione, Dulbecco’s Modified Eagle medium (DMEM), glutaraldehyde, sodium cacodylate, osmium tetroxide, potassium ferrocyanide, Spurr Low Viscosity Embedding kit, L-glutamine, tylosin, penicillin, and streptomycin were acquired from Sigma-Aldrich/Merck (São Paulo, Brazil). LIVE/DEAD^®^ BacLight™ kit was purchased from ThermoFisher Scientific (São Paulo, Brazil). Fetal bovine serum was acquired from Nova Biotecnologia (São Paulo, Brazil). Cation-Adjusted Mueller–Hinton Broth (CaMHB) was acquired from BD BBL™ (São Paulo, Brazil). Mueller–Hinton agar (MHA), Tryptone Soya Broth (TSB), and Tryptone Soya Agar (TSA) were acquired from HiMedia (Mumbai, India). Hydrogen peroxide (purity > 50%) was acquired from Dinâmica, Indaiatuba, São Paulo). Biogenic silver nanoparticles (bioAgNPs) were provided by GRAL Bioativos^®^ LDTA (Londrina, Brazil). The bioAgNP biosynthesis involved AgNO_3_ reduction using the aqueous extract of *Trichilia catigua* Adr. Juss bark [41]. For all antibacterial assays, GER was dissolved in 1% DMSO and 0.5% Tween^®^ 80 for the preparation of a stock solution (40 mg/mL). BioAgNPs were dissolved in ultrapure sterilized water to obtain a 1.08 mg/mL stock solution. The stock solutions were maintained at −20 °C and further diluted in the culture medium to achieve the concentrations used in each assay. The final concentrations of DMSO and Tween^®^ 80 did not exceed 0.5% and 0.25%, respectively, in any assay. The sterility control consisted of culture medium plus 0.5% DMSO and 0.25% Tween^®^ 80, whereas the growth control included culture medium plus 0.5% DMSO and 0.25% Tween^®^ 80, plus bacterial cells.

### 3.2. Bacterial Strains and Growth Conditions

Methicillin-resistant *Staphylococcus aureus* strains (n = 9) were selected from the bacterial collection of the Laboratory of Molecular Biology of Microorganisms, State University of Londrina, Londrina, Paraná, Brazil, according to their SCC*mec* types [3,4] (Supplementary Table S1). The MRSA strains were recovered from bloodstream infections and tissue fragments. The ST239/SCC*mec* III Brazilian Epidemic Clone (BEC) 9393 [70] was also included in the study. The bacterial strains were cultured on TSA at 37 °C for 24 h. To prepare the standard bacterial suspensions, three colonies of each strain were transferred into a 0.15 M NaCl solution (saline) to achieve turbidity equivalent to a 0.5 McFarland standard (1.0–2.0 × 10^8^ CFU/mL), using a DensiCHECK^™^ PLUS colorimeter (bioMérieux, Rio de Janeiro, Brazil). The standard bacterial suspensions were further diluted in culture medium to achieve the inoculum used in each assay. Bacteria were stored in TSB containing 15% glycerol at −80 °C.

### 3.3. Antibacterial Activity Against Planktonic Cells

#### 3.3.1. Minimum Inhibitory (MIC) and Minimum Bactericidal (MBC) Concentrations

The MICs of GER and bioAgNPs were determined by the broth microdilution assay, as recommended by the Clinical and Laboratory Standards Institute [71]. For these assays, the stock solutions of GER and bioAgNPs were serially diluted in CaMHB to achieve concentrations ranging from 19.53–10,000 μg/mL and 0.52–270 μg/mL, respectively. The MIC was defined as the lowest concentration capable of inhibiting visual bacterial growth after 24 h of incubation at 37 °C, compared to the growth control (without compounds). The antibacterial activity of GER was interpreted according to the following criteria: 0.05–0.5 mg/mL, strong activity; 0.6–1.5 mg/mL, moderate activity; and >1.5 mg/mL, inactive [36].

To estimate the MBC, wells with no visual growth were homogenized, and 10-µL aliquots were inoculated onto MHA and incubated at 37 °C for 24 h. The MBC was defined as the lowest concentration that inhibited 99.9% (3 log_10_) of CFU counts. The antibacterial effect was classified based on the MBC/MIC ratio as follows: bactericidal, MBC/MIC = 1–4; and bacteriostatic, MBC/MIC > 4 [37].

#### 3.3.2. Checkerboard Microdilution Assay

The antibacterial effect of GER combined with bioAgNPs was evaluated using the checkerboard broth microdilution assay, as described previously [38]. Two-fold serial dilutions of GER (4.88 to 625 µg/mL) and bioAgNPs (0.01 to 8.43 µg/mL) in CaMHB were added across the rows and columns, respectively, of U-bottom, 96-well microtiter plates (Techno Plastic Products, Trasadingen, Switzerland). Then, bacterial cells (1 × 10^5^ CFU/mL) were inoculated, and the microtiter plates were incubated at 37 °C for 24 h. The fractional inhibitory concentration (FIC) of each compound was determined by calculating the ratio of the MIC values of compounds tested in combination to the individual MIC. The FIC index (FICI) was subsequently calculated as the sum of the FIC values for geraniol (FIC_GER_) and biogenic silver nanoparticles (FIC_bioAgNPs_), and the values were interpreted as follows: synergistic, FICI ≤ 0.5; no interaction, 0.5 < FICI < 4.0; or antagonistic, FICI > 4.0 [39].

#### 3.3.3. Time–Kill Assay

The rate of MRSA killing in the presence of compounds, alone and in combination, was analyzed by the time–kill assay [72]. Approximately 1 × 10^5^ CFU/mL were added to 6 mL of CaMHB containing GER or bioAgNPs at their MIC or MBC, or the GER/bioAgNPs combination at synergistic concentrations. Bacterial growth in the absence of the compounds was used as the control. The cultures were incubated at 37 °C, and at specified time points (0, 4, 8, 12, and 24 h), CFU counts were determined. For this, a 10-µL aliquot was removed, serially diluted (1:10) in saline, and 10 µL of each dilution was inoculated onto MHA. The CFU counts were carried out after incubation at 37 °C for 24 h. Averaged data were plotted as log_10_ CFU/mL versus time (h). The bactericidal effect of the compounds was defined as a 99.9% (3 log_10_) reduction in CFU/mL compared to the initial inoculum [73].

### 3.4. Mode of Action of Compounds on Planktonic Cells

#### 3.4.1. Cell Viability

The effect of GER, alone or combined with bioAgNPs, on bacterial cell viability was evaluated using the LIVE/DEAD^®^ BacLight™ kit, according to the manufacturer’s recommendations. Approximately 1.5 × 10^8^ CFU/mL were inoculated into CaMHB containing the MIC values of GER and bioAgNPs alone or in combination, and at a synergistic concentration. The systems were incubated at 37 °C for 1 h. Afterward, untreated and treated bacterial cells were incubated with SYTO 9™ (6 μM) and propidium iodide (30 μM) at room temperature for 15 min. The cells were then observed under a fluorescence microscope (OLYMPUS BX53, Tokyo, Japan) using a fluorescein filter with excitation/emission wavelengths of 480/530 nm, respectively. The green-fluorescent nucleic acid stain SYTO 9™ labels live and dead bacteria, whereas the red-fluorescent nucleic acid stain propidium iodide selectively labels bacteria with permeable (damaged) membranes.

#### 3.4.2. Transmission Electron Microscopy (TEM)

Morphological alterations in MRSA planktonic cells treated with the compounds, alone or in combination, were analyzed using TEM. After incubation at 37 °C for 12 h, the cells were washed with phosphate-buffered saline (PBS), fixed in 2.5% glutaraldehyde in 0.1 M sodium cacodylate buffer, and post-fixed in a solution containing 1% osmium tetroxide, 0.8% potassium ferrocyanide, and 5 mM calcium chloride. The planktonic cells were dehydrated in an acetone series and embedded in Spurr resin for 72 h at 60 °C. Ultrathin sections were obtained in a Leica EM UC7 ultramicrotome (Leica Microsystems, Wetzlar, Germany) and stained with 5% uranyl acetate and lead citrate. TEM micrographs were acquired using a JEOL JEM-1400 transmission electron microscope (JEOL Ltd., Tokyo, Japan).

#### 3.4.3. Effect on Staphyloxanthin Biosynthesis

The effect of GER and bioAgNPs on staphyloxanthin biosynthesis was assessed as described in [44]. For qualitative analysis, MRSA was cultured on MHA containing subinhibitory concentrations of GER (156.25 μg/mL), bioAgNPs (0.52 μg/mL), or the GER/bioAgNPs combination (78.17/0.26 μg/mL) at 37 °C for 24 h. For quantitative analysis, the carotenoid pigment was extracted using methanol. Briefly, MRSA was grown in 1 mL of CaMHB containing subinhibitory concentrations of compounds, either alone or in combination, at 37 °C for 24 h. The cultures were centrifuged at 8000 rpm for 10 min; the cells were washed with 0.15 M PBS, pH 7.2, and suspended in 1 mL of methanol. The tubes were protected from light and incubated at 37 °C for 24 h under constant agitation (100 rpm). Afterward, the tubes were centrifuged, and the supernatant was used to quantify the extracted pigments by measuring the optical density (OD) at 462 nm using a BioTek Synergy^™^ HT microtiter plate reader (Agilient, Santa Clara, CA, USA). The percentage of inhibition was calculated using the following formula: % inhibition = [(Control OD_462nm_ − Treated OD_462nm_)/Control OD_462nm_] × 100. CaMHB without GER and CaMHB with 100 µg/mL thymol [44] were used as negative and positive controls, respectively, in both assays.

#### 3.4.4. Hydrogen Peroxide Killing Assay

The effect of the compounds on the susceptibility of MRSA to hydrogen peroxide was assessed as described in [44]. Bacterial cells were grown in CaMHB in the absence and presence of GER and bioAgNPS, alone or in combination, as detailed above. Then, the cell pellets were resuspended in 1 mL of PBS containing 0.2% hydrogen peroxide, and incubated at 37 °C for 3 h. After this period, 100 μL of each treatment were inoculated onto MHA, and CFU counts were carried out after incubation at 37 °C for 24 h.

### 3.5. In Silico Analysis

#### Molecular Docking

The structures of the dehydrosqualene synthase (CrtM; PDB ID: 3TFP, resolution 2.0 Å) and the aldehyde dehydrogenase (AldH; PDB ID: 6K10, resolution 1.79 Å) were prepared using the Discovery Studio software (BIOVIA, San Diego, CA, USA, version 21.1.0.20298), in which water molecules and crystallization artifacts were removed. For GER, tautomers were checked at pH 7.4 using the software MarvinSketch (Chemaxon, Budapest, Hungary, v. 23.8.0), and geometry was minimized using the MMFF94 force field, implemented in Avogrado (v. 1.2.0). CrtM presented a co-crystallized ligand in its active site (03L), enabling validation of the protocol by redocking. The coordinates used were anchored to the position of the co-crystallized ligand (X: −2.086414 Y: −28.960655 Z: 7.204586) at a radius of 10 Å. A total of ten iterative runs were carried out on each of the four scoring functions (ASP, ChemPLP, ChemScore, and GoldScore) in the Genetic Optimization for Ligand Docking (GOLD) software (CCDC, Cambridge, UK, version 2024.2.0), in the search of a function that yielded a root mean square deviation (RMSD) of less than 2 Å. The validated function was GoldScore (RMSD: 1.073 Å). For AldH, as there was no co-crystallized ligand, the molecular docking protocol was validated by consensus among the four functions in the software (RMSD ≤ 2.0 Å). The coordinates were anchored to the two internal amino acid residues of the catalytic pocket, Tyr116 and Ser244 (X: 8.035000 Y: 26.102500 Z: 103.988500), within a radius of 10 Å, with 10 iterative runs. Molecular ligand-target interactions were analyzed using Discovery Studio software (BIOVIA, San Diego, CA, USA, v. 21.1.0.20298) and PyMOL ((Schrödinger, Cambridge, MA, USA, v. 2.5.5).

### 3.6. Antibacterial Effect on Sessile (Biofilms) Cells

#### 3.6.1. Antibiofilm Activity of Geraniol and bioAgNPs, Alone or in Combination, on Pre-Formed Biofilms

Biofilms of MRSA strains were formed on flat-bottom, 96-well microtiter plates (Techno Plastic Products, Trasadingen, Switzerland) during 24 h, as described in [38], with minor modifications. A 20-µL aliquot of standard bacterial suspension was added into each well containing 180 µL of TSB supplemented with 1% glucose (TSB + G), and the plates were incubated statically at 37 °C for 24 h (Supplementary Figure S5). After incubation, the medium was aspirated, and non-adherent cells were removed by washing with PBS. Fresh medium (100 µL) containing different concentrations of GER (19.53–2500 μg/mL) or bioAgNPs (2.10–135 µg/mL) was added, and the biofilms were incubated at 37 °C for 24 h. Given that bioAgNPs do not interfere with the spectrophotometric analysis of the MTT reduction assay [74], this approach was utilized to analyze the effect of compounds on the metabolic activity of sessile cells. Following incubation, biofilms were washed with PBS, and the metabolic activity of sessile cells was estimated by the MTT reduction assay. A 100-µL aliquot of MTT (0.5 mg/mL)/menadione (0.5%) solution was added to each well, and the plates were incubated in the dark at 37 °C for 2 h. Formazan crystals were dissolved by the addition of 100 µL of solubilizing solution (10% Triton X-100, 0.1 N HCl in isopropanol) before spectrophotometric readings at 550 nm in a BioTek Synergy^™^ HT microtiter plate reader. The MIC was determined as the lowest concentration capable of inhibiting 90% of the metabolic activity of sessile cells (SMIC_90_) compared to the untreated controls.

To evaluate the effect of GER combined with bioAgNPs, biofilms were formed as described above, and after the removal of the non-adherent, two-fold serial dilutions of GER (9.76 to 1250 µg/mL) and bioAgNPs (0.13 to 67.5 µg/mL) were added across the rows and columns, respectively, of the 96-well microtiter plates. The SMIC_90_ of the GER/bioAgNPs combination was determined by measuring the metabolic activity of sessile cells using the MTT reduction assay, as detailed above. The FICI was calculated, and the values were interpreted using the same criteria applied to planktonic cells.

#### 3.6.2. Effect of Geraniol and bioAgNPs, Alone or in Combination, on Adhesion and Biofilm Formation

The effect of the compounds on MRSA adhesion to a polystyrene surface was determined as described by Liu et al. [75], with minor modifications. A 90-μL volume of TSB + G containing subinhibitory concentrations of GER (39.06 to 312.5 µg/mL) and bioAgNPs (0.52 to 4.21 µg/mL), either alone or in combination (9.76/0.03 to 78.12/0.26 µg/mL), was transferred to the wells of flat-bottom, 96-well microtiter plates. The standard bacterial suspension (10 μL) was added into the wells, and the plate was incubated at 37 °C for 4 h. Then, the wells were washed three times with PBS to remove non-adherent cells, and the metabolic activity of adherent cells was estimated by using the MTT reduction assay, as previously described.

The inhibitory effect of the compounds on biofilm formation was evaluated as described by [38], with minor modifications. After the adhesion of planktonic cells, non-adherent cells were removed by washing with PBS. Fresh TSB + G (200 µL), containing subinhibitory concentrations of GER (39.06 to 312.5 µg/mL) and bioAgNPs (0.52 to 4.21 µg/mL), alone or in combination (9.76/0.03 to 78.12/0.26 µg/mL), was added to the wells and incubated at 37 °C for an additional 24 h. The metabolic activity of sessile cells was also estimated using the MTT reduction assay.

#### 3.6.3. Scanning Electron Microscopy (SEM)

Morphological alterations in MRSA biofilms treated with the compounds or the combination were analyzed using SEM. Strips of polystyrene (1.0 cm^2^) were placed into the wells of 24-well cell culture plates containing 1 mL TSB + G inoculated with MRSA (1 × 10^7^ CFU), and the systems were incubated at 37 °C for 24 h. The biofilms were washed twice with PBS, and treated with SMIC_90_ of GER and bioAgNPs alone, or the GER/bioAgNPs combination at the synergistic concentration at 37 °C for 24 h. The biofilms were washed with PBS, then fixed with 2.5% (*v*/*v*) glutaraldehyde in 0.1 M sodium cacodylate buffer (pH 7.2) at room temperature for 4 h, dehydrated with serial ethanol washes (30%, 50%, 70%, 90%, and 100%), critical point-dried using CO_2_ (BAL-TEC, CPD 030), coated with gold, and observed in a FEI Quanta 250 scanning electron microscope (Thermo Fisher Scientific, Waltham, MA, USA).

### 3.7. Toxicity Analyses

#### 3.7.1. Effect of Geraniol and bioAgNPs, Alone or in Combination, on Mammalian Cells

The toxicity of compounds for HaCat cells (human keratinocyte cell line, CLS 300493, Heidelberg, Germany) was analyzed as described in [74]. Cells were cultured in flat-bottomed 96-well microtiter plates containing DMEM supplemented with 10% fetal bovine serum, 2 mM L-glutamine, 1% tylosin, 100 IU/mL penicillin, 100 µg/mL streptomycin, and 5% CO_2_ at 37 °C for 24 h. After incubation, non-adherent cells were removed by gently washing with sterile PBS. Culture medium containing GER (625 µg/mL), bioAgNPs (8.43 µg/mL), or GER/bioAgNPs (156.25/2.10 µg/mL) was added, and the cells were incubated for a further 24 h. Cell viability was analyzed by the MTT reduction assay, according to the manufacturer’s recommendation.

#### 3.7.2. Effect of Geraniol and bioAgNPs, Alone or in Combination, on *G. mellonella* Larvae

Seven groups of ten larvae (instar stage) without apparent color alterations and weighing between 200 to 300 mg were placed separately in Petri dishes. Larvae were cleaned with 70% ethanol, and 10 µL of PBS containing different concentrations of the compounds were injected into the larval hemocoel via the last left proleg using a Hamilton syringe (Merck, São Paulo, Brazil). The groups of larvae received the following compound concentrations per kg of larvae: 1250 and 2500 µg/mL of GER; 67.5 and 135 µg/mL of bioAgNPs; and 156.25/2.10 and 312.5/4.21 µg/mL of the GER/bioAgNPs combinations. Groups of larvae inoculated with PBS, and PBS plus 1% DMSO plus 0.5% Tween^®^ 80, were used as controls. The larvae were incubated at 37 °C in the dark, and survival was monitored every 24 h for up to 120 h. Larval death was assessed through visual analysis of melanization (presence of dark spots on their bodies) and the absence of movement in response to physical stimuli with forceps [74].

### 3.8. Statistical Analyses

All of the experiments were carried out in at least two biological replicates, on at least two different occasions, and the results were expressed as the mean ± standard deviation. Statistical analyses were performed using GraphPad PRISM software version 8.0 (GraphPad Software, San Diego, CA, USA). Differences between the control and treated groups were analyzed using one-way ANOVA, followed by Tukey’s multiple comparisons test. For all assays, *p* < 0.05 was considered statistically significant.

## 4. Conclusions

The present study reports the antibacterial activity of geraniol (GER), alone and combined with biogenic silver nanoparticles (bioAgNPs, synthesized via an aqueous extract of *Trichilia catigua* A. Juss. bark), against planktonic and sessile (biofilm) cells of methicillin-resistant *Staphylococcus aureus* (MRSA). The key findings include the following: (i) GER exhibits bactericidal activity against planktonic cells by impairing the cell membrane integrity; (ii) GER inhibits the staphyloxanthin production, an effect that may be attributed to the inhibition of the CrtM and AldH enzymes in the pigment biosynthesis pathway; (iii) the inhibition of staphyloxanthin production by GER may be related to the increased sensitivity of MRSA to hydrogen peroxide killing; (iv) GER exhibits synergism with bioAgNPs against planktonic MRSA cells, and, although the nature of this interaction was classified as bacteriostatic, it resulted in a four-fold reduction in MIC values of both compounds; (v) the GER/bioAgNPs combination also inhibits the adhesion of bacterial cells, biofilm formation, and pre-formed biofilms (24 h) on the polystyrene surface; and (vi) the GER/bioAgNPs combination does not cause toxicity to mammalian cells and *G. mellonella* larvae.

The following limitations of this study may restrict the generalization of our findings: the limited number of clinical isolates tested do not represent all of the antimicrobial susceptibility and genotypic profiles of MRSA; most of the assays were performed in vitro conditions; the mode of action of the combination has not yet been fully elucidated; and an insect model has been utilized for the toxicity assays. Despite these limitations, our results indicate that the GER/bioAgNPs combination may be a promising and safe starting point for an alternative or adjuvant therapy to control MRSA infections.

## Figures and Tables

**Table 1:** Antibacterial activity of GER and bioAgNPs, alone and in combination, against planktonic cells of methicillin-resistant *Staphylococcus aureus*.

*Staphylococcus aureus*	GER (µg/mL)			bioAgNPs (µg/mL)			GER/bioAgNPs ^b^ (µg/mL)	FICI ^c^	Interaction ^d^
	MIC	MBC	MBC/MIC ^a^	MIC	MBC	MBC/MIC ^a^			
BEC 9393	625	1250	2	8.43	16.87	2	156.25/2.10	0.5	Synergy
108	625	1250	2	8.43	16.87	2	156.25/2.10	0.5	Synergy
149	625	2500	4	8.43	16.87	2	156.25/2.10	0.5	Synergy
1	625	2500	4	8.43	16.87	2	156.25/2.10	0.5	Synergy
26	625	2500	4	8.43	16.87	2	156.25/2.10	0.5	Synergy
37	625	1250	2	8.43	16.87	2	156.25/2.10	0.5	Synergy
598	625	1250	2	8.43	16.87	2	156.25/2.10	0.5	Synergy
5	625	2500	4	8.43	16.87	2	156.25/2.10	0.5	Synergy
39	625	2500	4	8.43	16.87	2	156.25/2.10	0.5	Synergy
518	625	2500	4	8.43	16.87	2	156.25/2.10	0.5	Synergy

BEC: Brazilian epidemic clone; GER: geraniol; bioAgNPs: biogenic silver nanoparticles obtained from the reduction of silver nitrate using the aqueous extract of *Trichilia catigua* A. Juss. bark; MIC: minimum inhibitory concentration; MBC: minimum bactericidal concentration. **^a^** Reference values: 1 to 4 bactericidal effect and >4 bacteriostatic effects [37]. **^b^** MIC of GER and bioAgNPs in combination determined by checkerboard assay [38]. **^c^** FICI: fractional inhibitory concentration index was calculated as the sum of the FIC_GER_ and FIC_bioAgNPs_. **^d^** Reference values: synergism (FICI ≤ 0.5), no interaction (0.5 < FICI < 4.0), or antagonism (FICI > 4.0) [39].

**Table 2:** Antibiofilm activity of GER and bioAgNPs, alone and in combination, against sessile methicillin-resistant *Staphylococcus aureus*.

*Staphylococcus aureus*	SMIC_90_			FICI ^b^	Interaction ^c^
	GER (µg/mL)	bioAgNPs (µg/mL)	GER/bioAgNPs ^a^ (µg/mL)		
BEC 9393	1250	67.5	156.25/0.52	0.13	synergy
108	1250	67.5	156.25/0.26	0.13	synergy
149	625	16.87	156.25/4.21	0.49	synergy
1	1250	67.5	156.25/4.21	0.19	synergy
26	625	33.75	156.25/4.21	0.37	synergy
37	1250	67.5	156.25/0.52	0.13	synergy
598	1250	67.5	156.25/0.26	0.12	synergy
5	1250	67.5	156.25/1.05	0.13	synergy
39	625	67.5	156.25/8.43	0.37	synergy
518	1250	67.5	156.25/0.26	0.12	synergy

BEC: Brazilian epidemic clone; GER: geraniol; bioAgNPs: biogenic silver nanoparticles obtained from the reduction of silver nitrate using the aqueous extract of *Trichilia catigua* A. Juss. bark; SMIC_90_: sessile minimum inhibitory concentration capable of inhibiting 90% of the metabolic activity of sessile cells. ^a^ SMIC_90_ of GER and bioAgNPs in combination, determined by checkerboard assay [38]. ^b^ FICI: fractional inhibitory concentration index was calculated as the sum of the FIC_GER_ and FIC_bioAgNPs_. ^c^ Reference values: synergism (FICI ≤ 0.5), no interaction (0.5 < FICI < 4.0), or antagonism (FICI > 4.0) [39].

## Data Availability

The original contributions presented in this study are included in the article/Supplementary Materials. Further inquiries can be directed to the corresponding author.

## Supplementary Materials

The following supporting information can be downloaded at: www.mdpi.com/10.3390/plants14071059/s1, Table S1. Characteristics of methicillin-resistant *Staphylococcus aureus* used in this study; Figure S1. Effect of subinhibitory concentrations (sub-MIC) of geraniol (GER) and biogenic silver nanoparticles (bioAgNPs), alone or in combination, on the growth of MRSA BEC 9393; Figure S2. Overview of the staphyloxanthin biosynthesis pathway in *Staphylococcus aureus*; Figure S3. Structure and catalytic mechanism of CrtM (in purple). Figure S4. Effect of geraniol (GER) and biogenic silver nanoparticles (bioAgNP) on *Galleria mellonella* larvae. Figure S5. Temporal development of methicillin-resistant *Staphylococcus aureus* BEC 9393 biofilm on polystyrene surface.
