# Supplementary material for: Synergistic Antibacterial Interaction of Geraniol and Biogenic Silver Nanoparticles on Methicillin-Resistant Staphylococcus aureus
Source: Plants (Basel). 2025 Mar 29;14(7):1059. doi: 10.3390/plants14071059 (PMC11991589; doi:10.3390/plants14071059)
Supplement: Supplementary file 1 [file plants-14-01059-s001.zip › plants-3550015-supplementary.pdf]

Synergistic antibacterial interaction of geraniol and biogenic silver nanoparticles on methicillin-resistant *Staphylococcus aureus*

Isabela Madeira de Castro[1,2], Camila Antunes[2], Camila Cristina Valentim[2], Laís Fernanda de Almeida Spoladori[1,2], Helena Tiemi Suzukawa[1,2], Guilherme Ferreira Correia[1,2], Gislaine Silva-Rodrigues[1,2], Paulo Henrique Guilherme Borges[1,2], Guilherme Bartolomeu-Gonçalves[2,3], Mariana Luiza Silva[4], Marcelle de Lima Ferreira Bispo[4], Rayanne Regina Beltrame Machado[5], Celso Vataru Nakamura[1,5], Gerson Nakazato[1], Phileno Pinge-Filho[1,6], Eliandro Reis Tavares[1,2,7], Lucy Megumi Yamauchi[1,2] and Sueli Fumie Yamada-Ogatta[1,2,3,*]

Table S1: Characteristics of methicillin-resistant *Staphylococcus aureus* used in this study [3,4]

MRSA	Source	SCC*mec*	MIC vancomycin (µg/mL)
**BEC 9393**	Blood	III	1
**108**	Blood	I	1
**149**	Blood	I	1
**1**	Tissue fragment	II	4
**26**	Tissue fragment	II	2
**37**	Tissue fragment	III	4
**598**	Blood	III	1
**5**	Blood	IV	1
**39**	Tissue fragment	IV	4
**518**	Blood	VI	0.5

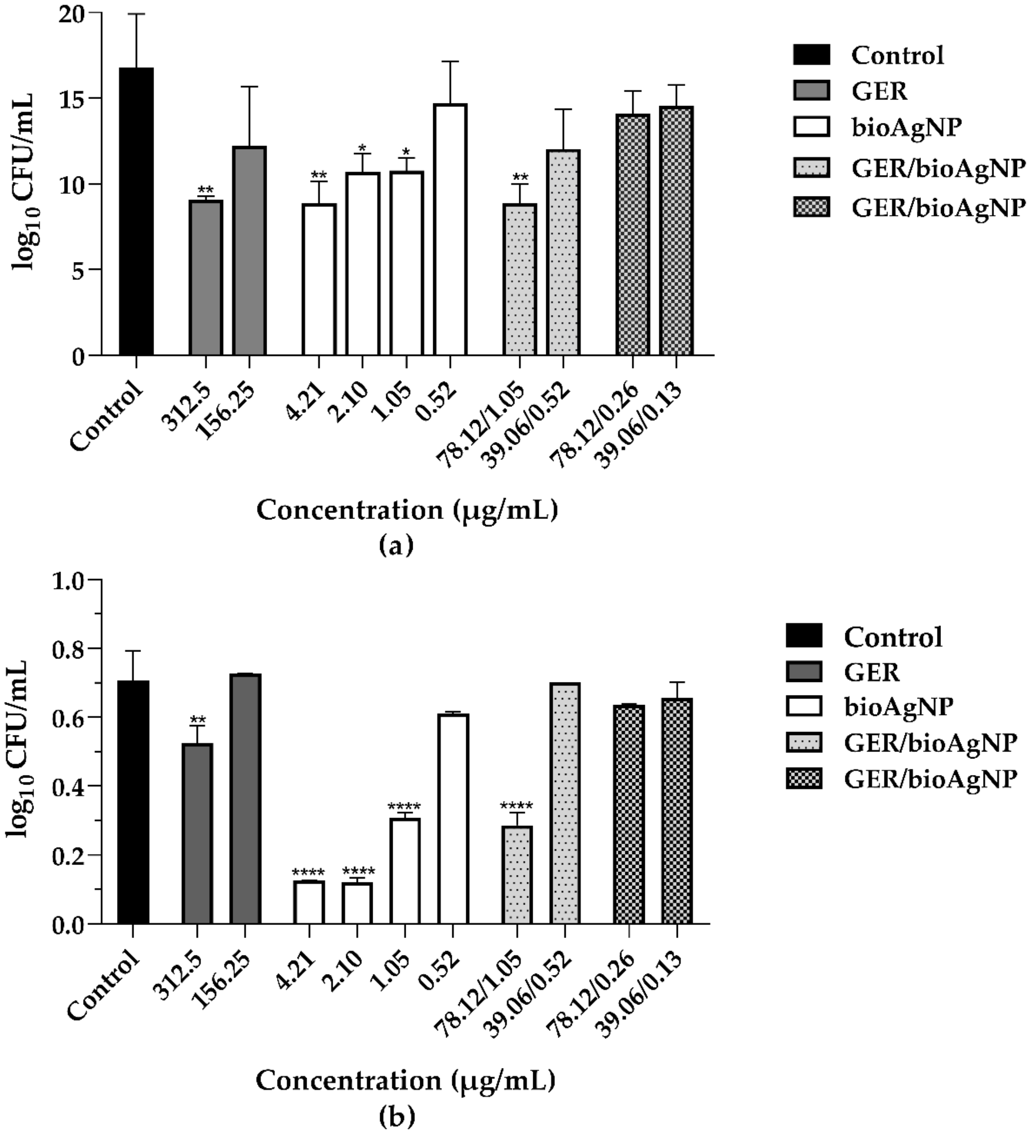

Figure S1: Effect of subinhibitory concentrations (sub-MIC) of geraniol (GER) and biogenic silver nanoparticles (bioAgNPs), alone or in combination, on the growth of MRSA BEC 9393. Planktonic cells were incubated in CaMHB containing sub-MICs of the compounds alone or their combination at 37 °C for 24 h. (**a**) Bacterial growth estimated by colony-forming units counts; (**b**) Bacterial growth estimated by optical density at 600 nm. *(p<0.05), **(p<0.01), ****(p<0.0001) compared with the control.

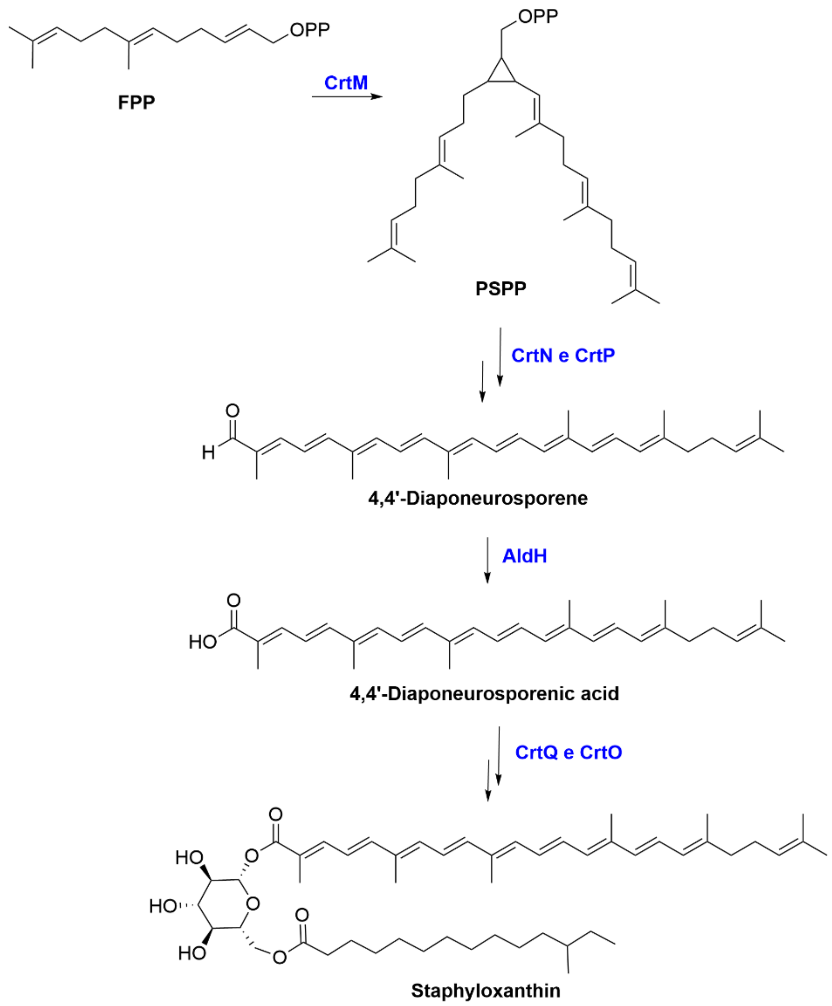

Figure S2: Overview of the staphyloxanthin biosynthesis pathway in *Staphylococcus aureus.* The staphyloxanthin production pathway is controlled by the *crtOPQMN* operon. Dehydrosqualene synthase (CrtM) catalyzes the condensation of two molecules of farnesyl diphosphate into dehydrosqualene, followed by the steps catalyzed by 4,4′-diapophytoene desaturase (CrtN), 4,4′-diaponeurosporene oxidase (CrtP), glycosyl-transferase CrtQ and acyltransferase CrtO, resulting in the formation of 4,4-diapophytoene, 4,4-diaponeutosporene, 4,4-diaponeurosporenic acid, glucosyl-4,4-diaponeurosporenic acid, and staphyloxanthin, respectively [54]. The 4,4′-diaponeurosporen-aldehyde dehydrogenase (AldH) of *S. aureus* catalyzes the oxidation reaction of 4,4′-diaponeurosporen-4-al into 4,4′-diaponeurosporenoic acid [55,56]. These enzymes promote functional group addition reactions and the formation of double bonds, resulting in the final structure of staphyloxanthin.

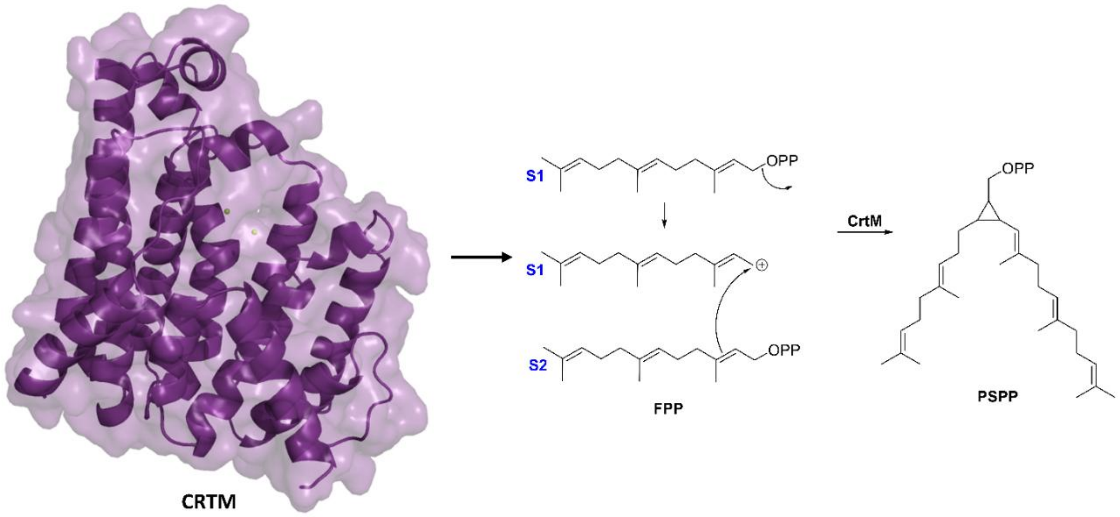

Figure S3. Structure and catalytic mechanism of CrtM (purple). The CrtM possesses two active sites for the conversion of farnesyl diphosphate (FPP) into presqualene diphosphate (PSPP), which is the precursor of staphyloxanthin. FPP binds to the site 1 (S1) and is ionized to form a primary carbocation. Magnesium ions and residues such as arginine and tyrosine are responsible for removing the carbocation-S1 complex. This carbocation then translocates to site 2 (S2) to react with the prenyl diphosphate present at this site to form PSPP [58].

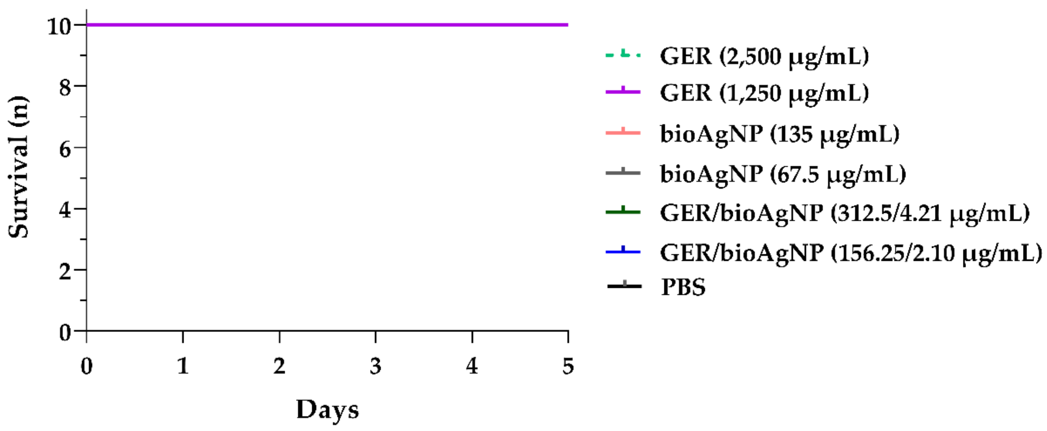

Figure S4. Effect of geraniol (GER) and biogenic silver nanoparticles (bioAgNPs) on *Galleria mellonella* larvae. Kaplan–Meier survival curves of *G. mellonella* larvae. The larvae were inoculated with GER and bioAgNPs alone, and GER/bioAgNPs combinations. The larval survival was monitored for 5 days.

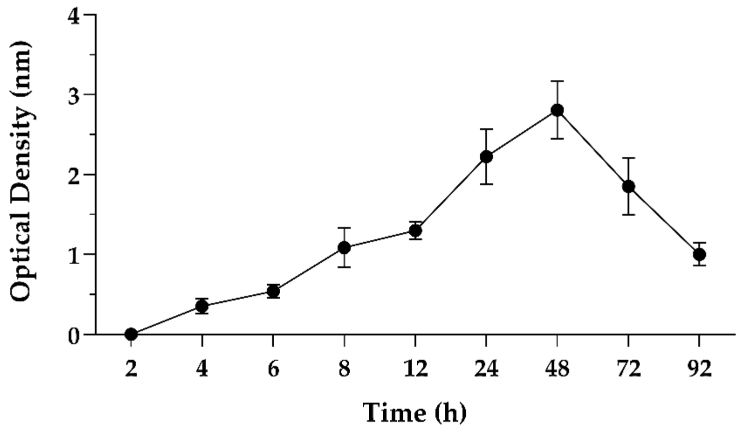

Figure S5. Temporal development of methicillin-resistant *Staphylococcus aureus* BEC 9393 on polystyrene surface. Biofilms were formed in TSB+G, and, at specified times, the metabolic activity of sessile cells was determined by the MTT reduction assay.
